# The Lysosome–Cathepsin Axis in Pancreatic Cancer: Mechanisms of Stromal Remodeling, Immune Evasion, and Therapy Resistance

**DOI:** 10.3390/biom16060824

**Published:** 2026-06-02

**Authors:** Nika Mazej Jeram, Emanuela Senjor, Janko Kos, Milica Perišić Nanut

**Affiliations:** 1 Department of Biotechnology, Jožef Stefan Institute, Jamova cesta 39, 1000 Ljubljana, Slovenia; 2 Faculty of Pharmacy, University of Ljubljana, Aškerčeva cesta 7, 1000 Ljubljana, Slovenia

**Keywords:** pancreatic ductal adenocarcinoma, lysosomal cathepsins, tumor microenvironment, immune evasion, extracellular matrix remodeling, autophagy, therapeutic resistance, cytotoxic cells, antigen presentation

## Abstract

Pancreatic cancer remains one of the most lethal malignancies worldwide, with pancreatic ductal adenocarcinoma accounting for the vast majority of cases and characterized by extensive desmoplasia, immune exclusion, and resistance to systemic therapies. Increasing evidence implicates lysosomal cathepsins as important regulators of these defining features of pancreatic tumor biology. Cathepsin-dependent proteolysis and lysosome-associated signaling pathways contribute to extracellular matrix remodeling, regulate immune cell trafficking, and influence antigen processing and presentation. Beyond their classical degradative functions, cathepsins participate in stress-adaptive cellular programs linked to autophagy, metabolic regulation, and proteostasis, supporting tumor cell survival under hypoxic, nutrient-limited, and therapy-induced stress conditions. Within the tumor microenvironment, dysregulated cathepsin activity promotes immune evasion by reshaping cytokine networks, impairing effective antigen presentation, and reinforcing physical and functional barriers to cytotoxic T-cell infiltration. Collectively, these mechanisms position the lysosome–cathepsin system as a central regulator of proteolytic remodeling, immune exclusion, and adaptive therapy resistance in pancreatic cancer, highlighting its potential relevance for emerging combinatorial therapeutic strategies.

## 1. Introduction

Pancreatic cancer remains one of the deadliest malignancies, with high mortality due to late diagnosis, early metastatic spread, and limited durable responses to current therapies. Pancreatic ductal adenocarcinoma (PDAC) is the most common and aggressive subtype, accounting for approximately 90% of cases, and still has a five-year survival rate below 12% [1]. Because early symptoms are non-specific and screening is ineffective, late diagnosis is typical, with most patients presenting with advanced, often unresectable tumors. Even when surgery is possible, relapse is common, and long-term survival remains poor, highlighting limited treatment progress and the urgent need for more effective, mechanism-driven therapeutic strategies [2,3].

PDAC is also among the most therapy-resistant cancers in clinical practice. For resectable disease, surgery followed by adjuvant chemotherapy (gemcitabine-based regimens or FOLFIRINOX) is standard; however, many patients never fully recover to receive treatment, and those who do often experience only modest benefit. In the palliative setting, response rates remain limited (8–32%) [4]. Resistance arises from both tumor-intrinsic mechanisms, such as KRAS-driven signaling, cancer stemness, and adaptive genetic or epigenetic rewiring, as well as from strong extrinsic pressures in the tumor microenvironment (TME) [5,6,7]. Dense desmoplasia, heterogeneous cancer-associated fibroblast (CAF) populations, immunosuppressive infiltrates, and metabolic reprogramming impair drug delivery, weaken anti-tumor immunity, and support tumor survival [8,9,10,11,12].

A major unmet need in PDAC is the consistent failure of immunotherapy. Unlike several other solid tumors, PDAC has shown poor and largely disappointing responses to immune checkpoint blockade and other immunotherapeutic strategies [13]. PDAC is historically considered an immunologically “cold” tumor, characterized by limited cytotoxic T-cell infiltration due to dense desmoplastic stroma, defective antigen presentation, and a highly immunosuppressive TME enriched in myeloid cells and CAFs. Notably, surface major histocompatibility complex (MHC) class I expression is often reduced despite the absence of recurrent inactivating mutations in core MHC-I genes, indicating that post-transcriptional mechanisms, such as autophagy-mediated MHC-I degradation, may contribute to immune evasion [14]. In this context, lysosomes and autophagy are gaining increasing attention as central regulators of tumor fitness and immune evasion in PDAC. Beyond their degradative role, lysosomes function as key hubs for metabolic signaling, coordinating pathways such as mTORC1 and transcription factor EB (TFEB) while serving as the terminal compartment for autophagic flux [15]. PDAC cells frequently exhibit elevated autophagic activity and a strong dependence on lysosomal function to buffer nutrient deprivation and oxidative stress, recycle macromolecules, and survive chemotherapy-induced damage. These adaptive processes support metabolic plasticity and stress tolerance and may indirectly reinforce immune escape by sustaining tumor cell viability within the nutrient-poor and immune-excluded PDAC TME [16,17]. Within this framework, lysosomal peptidases, particularly cathepsins, emerge as important effectors of lysosomal activity, linking intracellular protein turnover with extracellular matrix (ECM) remodeling, immune regulation, and tumor progression in PDAC [18,19,20].

This review systematically elucidates how lysosomal cathepsins influence PDAC progression, antitumor immune dysfunction, and therapy resistance. We interpret the lysosome–cathepsin axis as a PDAC-specific adaptive stress network rather than as a collection of isolated protease functions. Within this framework, oncogenic KRAS signaling, nutrient limitation, hypoxia, desmoplastic stiffness, inflammatory signaling, and therapy-induced stress converge on lysosomal biogenesis, autophagy, vesicular trafficking, cathepsin maturation and activation, and protease secretion. These processes connect intracellular metabolic adaptation with ECM remodeling, immune exclusion, altered antigen presentation, macrophage polarization, and treatment resistance across tumor-cell-intrinsic and microenvironmental compartments, including tumor-associated macrophages (TAMs), CAFs, and other stromal or immune populations. By positioning cathepsins at the intersection of lysosomal stress adaptation, stromal remodeling, and immune dysfunction, we outline mechanistically grounded opportunities for combinatorial therapeutic intervention. We also distinguish throughout the review between mechanisms directly demonstrated in PDAC and mechanisms inferred from other tumor systems or broader lysosomal biology.

## 2. Lysosomes and the Tumor Microenvironment

### 2.1. Lysosomal Degradation and Signaling Networks in Cellular and Tumor Homeostasis

Lysosomes are dynamic, membrane-bound acidic organelles containing approximately 60 hydrolytic enzymes, including proteases, lipases, and nucleases, responsible for degrading endocytic and autophagic cargo [21]. Once considered primarily terminal degradative compartments, lysosomes are now recognized as central regulators of cellular homeostasis, integrating proteolysis, metabolism, membrane trafficking, and signaling [22,23].

A principal pathway for delivering cytoplasmic material to lysosomes is macroautophagy (hereafter autophagy), a conserved catabolic process that maintains cellular integrity under both basal and stress conditions. Autophagy begins with the formation of a crescent-shaped isolation membrane (phagophore), orchestrated by autophagy-related (ATG) proteins. Progressive expansion and closure of the phagophore generate a double-membraned autophagosome that engulfs damaged organelles, protein aggregates, and other cytoplasmic components. Subsequent fusion with late endosomes and lysosomes forms autolysosomes, where lysosomal hydrolases, including cysteine and aspartic cathepsins, mediate cargo degradation. The resulting metabolites are recycled to sustain biosynthesis, ATP production, and redox balance. In cancer, sustained autophagic–lysosomal flux often confers a survival advantage by enabling tumor cells to withstand hypoxia, nutrient deprivation, and therapy-induced stress [24,25].

Beyond degradation and recycling, lysosomes function as nutrient-sensing and signaling hubs. The lysosomal surface serves as a platform for mTORC1 activation via Rag GTPases, coupling amino acid availability to anabolic growth programs. Under nutrient-replete conditions, active mTORC1 suppresses autophagy and promotes biosynthesis, whereas nutrient limitation inactivates mTORC1, permitting autophagic induction and lysosome-dependent recycling. TFEB coordinates this adaptive response by regulating the expression of genes involved in lysosomal biogenesis, autophagy, and vesicular trafficking. Through TFEB-dependent programs, the autophagy–lysosome axis dynamically adjusts degradative capacity to metabolic demand [15].

In addition to autophagy, macropinocytosis is a complementary route by which extracellular material is delivered to the lysosomal system. Macropinocytosis is an actin-driven endocytic process that enables bulk uptake of extracellular fluid, soluble antigens, and proteins, particularly in dendritic cells and macrophages [26,27]. After internalization, macropinosomes undergo a regulated maturation sequence characterized by Rab GTPase exchange, progressive acidification, and fusion with late endosomes and lysosomes [28]. Within these compartments, lysosomal hydrolases, including cathepsins, process internalized proteins into peptide fragments. In professional antigen-presenting cells (APC), this proteolysis supports loading of peptides onto MHC class II molecules, enabling CD4^+^ T-cell activation [29]. Under certain conditions, including antigen escape into the cytosol or delayed endosomal maturation in specialized dendritic cell subsets, macropinocytosed cargo can also enter cross-presentation pathways, linking antigen processing to MHC class I presentation and CD8^+^ T-cell priming [30,31,32]. Through these mechanisms, lysosomes couple environmental sampling to adaptive immune activation, reinforcing their role as dynamic regulators of immune homeostasis.

Beyond intracellular degradation, lysosomes also participate in membrane repair, vesicular trafficking, and regulated secretion, functions that are particularly relevant in the TME [33]. Importantly, lysosomal cargo is not confined to the cell interior. Lysosomal enzymes and partially processed substrates can reach the extracellular space through several routes: (i) lysosomal exocytosis, involving fusion of lysosomes with the plasma membrane; (ii) secretion via extracellular vesicles derived from multivesicular bodies with lysosome-related trafficking; and (iii) release following lysosomal membrane permeabilization or cell stress. In a protease-rich and metabolically stressed tumor niche, these export pathways extend lysosomal activity beyond the intracellular compartment, promoting ECM remodeling and modulating immune signaling [34].

PDAC exploits these lysosome-centered processes to adapt to its hostile TME, characterized by hypoxia, tissue acidosis, and nutrient limitation. Elevated autophagic flux, enhanced macropinocytosis, and altered proteostasis collectively converge on lysosomal degradation to sustain metabolic fitness and survival [35,36]. Consistent with this lysosomal dependency, increased lysosomal biogenesis and expansion of the lysosomal compartment have been reported in PDAC compared with normal pancreatic tissue [37], underscoring the central role of lysosomes as both metabolic hubs and platforms for extracellular proteolytic activity in this malignancy.

### 2.2. Unique Features of PDAC TME That Impact Lysosomal Function

**Figure 1:**
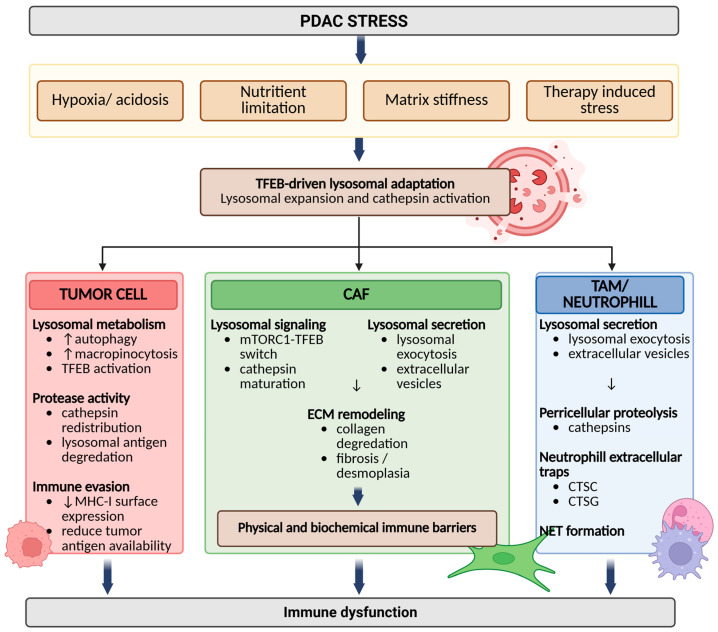
The PDAC TME Reprograms the Lysosome–Cathepsin Axis. PDAC is characterized by various stress conditions, including hypoxia, acidosis, nutrient limitation, and matrix stiffness associated with desmoplasia, as well as therapy-induced pressure. In tumor cells, CAFs and TAMs, these cues converge to activate lysosomal programs. This reprogramming is reflected by increased autophagic flux, enhanced macropinocytic trafficking to lysosomes, modulation of the mTORC1–TFEB axis with TFEB-driven lysosomal biogenesis, and proteolytic maturation of cathepsins from inactive pro-forms to active enzymes, with cystatins contributing to endogenous regulation of peptidase activity. Simultaneously, increased lysosomal exocytosis and extracellular vesicle release, along with acidification of the TME, facilitate extracellular activity of cathepsins such as cathepsin B (CTSB), cathepsin L (CTSL), and cathepsin D (CTSD). The resulting proteolytic remodeling of the ECM supports invasive growth, sustains desmoplastic reinforcement through CAF–TAM feedback circuits, and contributes to immune exclusion marked by limited T-cell and NK-cell infiltration. (Created with https://BioRender.com).

PDAC is characterized by an exceptionally harsh TME that strongly influences lysosomal activity (see Figure 1). A defining feature is the dense fibroinflammatory (desmoplastic) stroma, which can make up most of the tumor mass—on average about 60%, and up to 90% in some cases [35,36]. This ECM–rich compartment, composed largely of collagens and hyaluronan, increases solid stress and interstitial fluid pressure, compresses intratumoral vasculature, and restricts perfusion, creating a major physical barrier within the tumor [35,37,38]. Pancreatic stellate cells (PSCs), a principal source of CAFs, drive this desmoplastic reaction through extensive ECM deposition and bidirectional signaling with tumor cells, promoting tumor progression [39].

The PDAC stroma resembles chronic, non-resolving wound repair, characterized by persistent CAF activation, excessive ECM accumulation, and sustained inflammatory signaling [40]. Among stromal populations, α-SMA^+^ myofibroblast-like CAFs are particularly abundant and contribute to both matrix production and pro-tumorigenic signaling networks [41,42,43]. In addition to forming a structural barrier, CAFs and myeloid cells promote immune suppression through cytokines such as TGF-β and IL-6, leading to T-cell exclusion and therapy resistance [44,45,46]. These stromal and immune populations are also major sources of extracellular peptidases, including cathepsins, which participate in ECM remodeling and modulation of immune–stromal interactions within the PDAC TME [34,47].

#### 2.2.1. Hypoxia and Low pH (Turning Lysosomal Programs Outward)

Chronic hypoxia, caused by poor perfusion and high metabolic demand, is a defining feature of the PDAC TME. Hypoxia-driven metabolic rewiring enhances glycolysis and lactate export, leading to extracellular acidosis [48]. This acidic environment is particularly relevant for cathepsins, as many retain or even increase their proteolytic activity at low pH once released into the extracellular space. Notably, cathepsin B (CTSB; UniProt ID: P07858) and cathepsin L (CTSL; also known as cathepsin L1; UniProt ID: P07711) achieve optimal catalytic activity within acidic endolysosomal compartments (approximately pH 4.5–5.5), yet remain functionally active in the moderately acidic extracellular milieu characteristic of PDAC, where pH commonly decreases to around 6.5–6.8 in hypoxic and poorly perfused tumor regions. Although this extracellular pH is less acidic than that of the lysosomal lumen, localized pericellular acidification, interactions with cell-surface or ECM components, and sustained secretion of active enzymes may collectively support persistent CTSB- and CTSL-mediated proteolysis within the TME [47,49,50]. In parallel, hypoxia can further enhance cathepsin expression and proteolytic potential through HIF-1α–dependent transcriptional programs. Hypoxia and acidosis also promote secretory trafficking pathways, including extracellular vesicle release and redistribution of lysosomal contents into the TME, thereby extending lysosome-associated proteolysis beyond the intracellular compartment [51,52].

#### 2.2.2. Nutrient Limitation (Autophagy and Scavenging Through Lysosomes)

Limited vascularization in PDAC restricts nutrient delivery, forcing tumor cells to rely heavily on lysosome-dependent recycling pathways. Nutrient limitation within the PDAC TME is measurable and functionally restrictive, driving reliance on stress-adaptive metabolism [53]. Under these conditions, sustained autophagic flux and lysosomal turnover maintain intracellular amino acid and lipid pools when glucose and other nutrients are scarce [54]. PDAC cells also exploit macropinocytosis to scavenge extracellular proteins, which are subsequently degraded in lysosomes to supply metabolic substrates [55]. In Ras-driven tumors, oncogenic Ras promotes constitutive macropinocytosis, enabling tumor cells to internalize extracellular proteins that are degraded in lysosomes to generate amino acids and support anabolic metabolism under nutrient-limited conditions [32]. These metabolic constraints also affect immune cells within the TME: hypoxia and acidosis impair cytotoxic lymphocyte function and reinforce immunosuppressive programs, while lysosomal pathways in myeloid cells influence antigen processing and inflammatory signaling [49].

### 2.3. Lysosomal–Immune System Interface (Antigen Presentation, Cytokine Release, Cell Death)

Lysosomes serve as critical immune regulatory hubs that integrate antigen processing, inflammatory signaling, and stress responses. At this interface, lysosomal cysteine cathepsins play key roles by shaping antigen presentation, modulating inflammatory signaling, and influencing whether stressed cells undergo survival or inflammatory cell death [56]. In PDAC models, MHC-I molecules can be redirected into autophagosomes or lysosomes, reducing their surface presentation; inhibition of autophagy or lysosomal function restores MHC-I expression and enhances CD8^+^ T-cell–mediated tumor control [56]. In PDAC, impaired MHC-I antigen presentation is a hallmark of the tumor immune TME and contributes to its “immune-cold” phenotype. This is not primarily driven by recurrent genetic loss of MHC-I components, but rather by functional dysregulation of antigen processing pathways, including altered lysosomal trafficking, autophagy-dependent sequestration of MHC-I molecules, and impaired cross-presentation in dendritic cells. Additionally, lysosomal stress and metabolic reprogramming within PDAC further compromise antigen processing efficiency, limiting effective CD8^+^ T-cell activation and facilitating immune evasion [57]. These findings link metabolic stress programs, including nutrient limitation–induced autophagy, to immune evasion mechanisms in PDAC [56,57,58,59]. Consistently, PDAC frequently shows reduced MHC-I expression without recurrent inactivating mutations in core MHC-I genes and exhibits poor responsiveness to immune checkpoint blockade, further supporting a connection between metabolic adaptation and immune escape [56,60]. Beyond antigen presentation, lysosomes regulate cytokine release and inflammatory signaling. Several cytokines, including IL-1β and IL-18, can be secreted through lysosome-dependent unconventional pathways following inflammasome activation, processes associated with lysosomal destabilization and regulated exocytosis [61,62,63,64]. Lysosomal pathways also intersect with neutrophil extracellular trap (NET) formation. NETosis involves the mobilization of proteases from neutrophil azurophilic granules, lysosome-related organelles containing enzymes such as neutrophil elastase, myeloperoxidase, and cathepsin G (CTSG; UniProt ID: P08311), which translocate to the nucleus to promote chromatin decondensation and extracellular DNA release. In tumors, signals such as G-CSF, IL-8, and hypoxia stimulate NET formation, which can trap circulating tumor cells, remodel the ECM, and promote immunosuppressive and pro-thrombotic conditions within the TME [65]. In PDAC, NETs further contribute to stromal activation and metastatic spread [66]. Finally, lysosomal membrane permeabilization can release lysosomal peptidases into the cytosol, where they initiate or amplify apoptotic and inflammatory cell-death pathways in a context-dependent manner [67,68]. Together, these mechanisms position lysosomes as central regulators linking metabolic stress, immune signaling, and cell-fate decisions within the PDAC TME.

## 3. Cathepsins as Lysosomal Effectors in Pancreatic Cancer

### 3.1. Overview of Cathepsins

Cathepsins are lysosomal peptidases classified as cysteine, aspartic, or serine peptidases based on their catalytic mechanism. The human genome encodes eleven cysteine cathepsins (B, C, F, H, K, L, O, S, V, W, X/Z), two aspartic cathepsins (D, E), and two serine cathepsins (A, G) [69,70]. Cathepsins are synthesized as inactive zymogens and are activated mainly in acidic endolysosomal compartments by propeptide removal through autocatalysis or processing by other cathepsins [70,71].

Cathepsins differ in proteolytic specificity and catalytic mode. Most cathepsins function as endopeptidases, while cathepsin C (CTSC; also known as dipeptidyl peptidase I, DPP1; UniProt ID: P53634) is a dipeptidyl peptidase, cathepsin H (CTSH; UniProt ID: P09668) has aminopeptidase activity, and cathepsin Z/X (CTSZ/X; UniProt ID: Q9UBR2) functions as a carboxypeptidase [72]. CTSB exhibits both endo- and exopeptidase activity due to its unique occluding loop structure [73]. This functional diversity is important in PDAC, where individual cathepsins should not be interpreted as interchangeable markers of lysosomal activity, but rather as compartment- and context-dependent effectors of tumor invasion, metabolic adaptation, immune regulation, and biomarker biology.

Because the strength of evidence varies substantially among cathepsin-related mechanisms, we distinguish three levels of support throughout this review: (i) mechanisms directly demonstrated in PDAC models or patient-derived PDAC material, such as KRAS/TFEB-associated lysosomal remodeling, CTSB/CTSL-associated invasion, cathepsin D-associated gemcitabine resistance, and autophagy-dependent MHC-I degradation; (ii) mechanisms supported by PDAC-associated observations but not yet fully validated causally in human PDAC, such as cathepsin S-dependent immune modulation, cathepsin Z/X-associated integrin signaling, and stromal-mechanics-dependent YAP1 regulation; and (iii) mechanisms inferred from broader cancer or lysosomal biology, including selected ferroptosis-associated CTSB functions and some hypoxia- or cytokine-driven transcriptional pathways. This hierarchy is used to avoid overinterpreting associative, preclinical, or context-dependent findings.

### 3.2. Lysosomal Localization, Trafficking, and Activation Control of Cathepsin Activity

Cathepsins are synthesized as inactive zymogens whose activity is tightly regulated by intracellular trafficking, compartmental maturation, and endogenous inhibitors. They are translated on rough endoplasmic reticulum–bound ribosomes as preproenzymes, after which the signal peptide is removed and the nascent proenzymes undergo post-translational modification. N-linked glycosylation facilitates correct folding and lysosomal targeting while maintaining catalytic latency during biosynthesis and transport [70,74]. In the Golgi apparatus, most pro-cathepsins acquire mannose-6-phosphate (M6P) residues that enable recognition by mannose-6-phosphate receptors and delivery to endosomal compartments, although alternative receptor-independent trafficking pathways also exist [75].

Along the endosomal maturation pathway, pro-cathepsins enter progressively acidified compartments where receptor dissociation coincides with conditions that permit enzymatic activation. Maturation occurs primarily in late endosomes and lysosomes, where acidic pH destabilizes interactions between the propeptide and catalytic domain, enabling proteolytic removal of the inhibitory propeptide. Many cysteine and aspartic cathepsins undergo autocatalytic activation, while trans-activation by pre-existing active cathepsins further amplifies proteolysis within the lysosomal lumen [50,74].

Importantly, cathepsin activity is not determined solely by gene or protein expression levels. Instead, net proteolytic activity depends on intracellular routing, lysosomal pH, maturation state, secretion, substrate accessibility, and inhibition by endogenous inhibitors such as cystatins. Members of the cystatin family, including cystatin C, cystatin B, and related intracellular inhibitors, bind active cathepsins with high affinity and serve as key physiological brakes on proteolytic activity. Thus, the balance between cathepsins and their endogenous inhibitors critically determines the net proteolytic capacity of a cell [76]. Distinct pH and stability profiles further restrict activity along the endolysosomal continuum: enzymes such as cathepsin S (CTSS; UniProt ID: P25774), CTSC, and CTSH retain activity in late endosomes, whereas CTSB, cathepsin D (CTSD; UniProt ID: P07339), CTSL and cathepsin K (CTSK; UniProt ID: P43235) achieve maximal catalytic efficiency in fully acidified lysosomes [67]. In PDAC, oncogenic signaling and microenvironmental stress promote lysosomal biogenesis and increased lysosomal flux, expanding the pool of mature and catalytically competent cathepsins [76,77]. Alterations in lysosomal homeostasis or in the availability of endogenous inhibitors can further shift the balance toward sustained proteolysis, enhancing cargo degradation and supporting metabolic adaptation. Cathepsin regulation also intersects with TFEB-dependent lysosomal gene programs and autophagy. In PDAC models with impaired autophagy, dysregulated cathepsin activity is accompanied by increased extracellular cathepsin release and enhanced invasive behavior, underscoring that trafficking, maturation, and inhibitor balance, rather than expression alone, ultimately determine functional cathepsin output [77,78].

Multiple cathepsins are upregulated in PDAC compared with normal pancreatic tissue and are expressed across malignant epithelial, stromal, and immune compartments. Rather than representing a uniform increase in lysosomal enzymes, this pattern is best interpreted as part of an adaptive lysosomal state shaped by oncogenic KRAS signaling, TFEB-dependent lysosomal biogenesis, stromal stress, hypoxia, inflammation, and therapy-induced autophagy. The strongest PDAC-specific evidence supports KRAS–MAPK- and TFEB/MiT-TFE–dependent lysosomal remodeling, whereas direct evidence for HIF-1α- or NF-κB-mediated transcriptional regulation of cathepsins in PDAC remains comparatively limited.

Among these, cysteine cathepsins, such as CTSB, CTSL, and CTSS, promote tumor progression by remodeling the ECM and cleaving adhesion molecules, including E-cadherin, thereby facilitating invasion and dissemination. Genetic ablation of these enzymes reduces invasive growth in PDAC models [79]. Additional family members contribute to distinct aspects of tumor biology: CTSZ/X regulates cell–matrix interactions in tumor cells and macrophages, CTSH participates in pericellular proteolysis, and cathepsin W (CTSW; UniProt ID: P56202) is predominantly expressed in cytotoxic lymphocytes and reflects immune rather than tumor-intrinsic activity [80,81,82]. Aspartic cathepsins also participate in PDAC pathobiology: CTSD is frequently overexpressed and secreted by tumor cells and can be induced by oncogenic regulators such as anterior gradient protein 2 (AGR2), a protein disulfide isomerase family member involved in protein folding and regulation of the secretory pathway, whereas cathepsin E (CTSE; UniProt ID: P14091) is strongly upregulated in pancreatic intraepithelial neoplasia and invasive PDAC, consistent with early lysosomal reprogramming during tumorigenesis [83,84,85,86,87]. Recent experimental studies suggest that stromal mechanics may influence lysosomal cathepsin activity and YAP1 stability; however, these findings currently derive primarily from selected preclinical systems, and their broader relevance across heterogeneous human PDAC remains incompletely validated [88,89].

The term “lysosome–cathepsin axis,” as used in this article, refers to the integrated network connecting lysosomal dynamics with cathepsin-mediated proteolytic and signaling functions. It encompasses lysosomal biogenesis, vesicular trafficking, acidification, cathepsin maturation and activation, substrate degradation, and downstream signaling pathways regulated by lysosomal activity. Beyond their degradative role, lysosomes serve as signaling hubs that coordinate autophagy, nutrient sensing, inflammation, metabolic adaptation, and cell death pathways through interactions with mTORC1, TFEB/TFE3, inflammasomes, and mitochondrial quality-control mechanisms. Within this framework, cathepsins also regulate ECM remodeling, immune responses, apoptosis, and lysosomal stress signaling under physiological and pathological conditions [62,70]. Throughout this review, cathepsin biology is therefore discussed within an integrated PDAC-specific lysosomal framework rather than as isolated enzyme-specific observations.

### 3.3. Molecular Regulation of Cathepsin Expression in PDAC

Cathepsin dysregulation in PDAC appears to result from convergent oncogenic, metabolic, and microenvironmental signaling pathways that collectively reshape lysosomal function and proteolytic activity (see Table 1). The strongest PDAC-specific evidence supports a lysosome-centered regulatory model in which KRAS–MAPK signaling, MiT/TFE–TFEB activation, and therapy-induced autophagy coordinately expand lysosomal biogenesis and proteolytic capacity. In contrast, direct promoter-level evidence for HIF-1α-, NF-κB-, STAT3/STAT6-, Sp1-, miRNA-, or DNA methylation–dependent regulation of individual cathepsins in PDAC remains comparatively limited. Thus, cathepsin regulation in PDAC is better understood as coordinated remodeling of the autophagy–lysosome system, including lysosomal biogenesis, maturation, trafficking, secretion, and adaptive stress responses [90,91,92,93,94]. In this context, cathepsin abundance and activity are closely integrated with KRAS-driven metabolic rewiring, therapy-induced autophagy, hypoxic adaptation, and stromal signaling within the TME.

One of the best-characterized regulatory mechanisms in PDAC is activation of the MiT/TFE transcriptional network, particularly TFEB-dependent lysosomal programs. Perera et al. demonstrated that pancreatic cancer cells exhibit constitutive nuclear localization of MiT/TFE family members, resulting in sustained activation of a broad autophagy–lysosome transcriptional program that supports nutrient scavenging and tumor growth [90]. This lysosome-centered transcriptional state appears to be a fundamental adaptive mechanism in PDAC rather than a simple stress response. Subsequent studies demonstrated that therapeutic stress can further amplify this lysosomal program. MAPK pathway inhibition induces TFEB nuclear translocation, lysosomal biogenesis, and lysosomal drug sequestration, thereby contributing to adaptive resistance to MEK inhibition [91]. Similarly, gemcitabine promotes ERK-dependent autophagy and TFEB-mediated lysosomal expansion accompanied by increased CTSB activity [92]. Collectively, these findings indicate that oncogenic KRAS signaling and therapy-induced stress converge on TFEB-dependent lysosomal remodeling, thereby increasing lysosomal capacity, cathepsin maturation, and adaptive metabolic fitness in PDAC cells.

Additional evidence indicates that KRAS-associated lysosomal regulation also influences cathepsin trafficking and extracellular proteolytic activity. Gutierrez-Ruiz et al. identified dedicator of cytokinesis 8 (DOCK8) as a mediator of KRAS-dependent lysosomal remodeling in a subset of PDAC tumors [93]. DOCK8 promoted lysosomal motility, increased CTSB proteolytic activity, and enhanced cathepsin-dependent extracellular matrix degradation and invasion. These observations support the concept that oncogenic signaling regulates cathepsin function not only through expression, but also through lysosomal positioning, maturation, secretion, and extracellular trafficking. Consistent with this, CSTB has been shown to sustain CTSB proteolytic activity and autophagic flux, thereby coupling lysosomal proteolysis to glycolytic metabolism and metabolic fitness in PDAC cells [94].

Microenvironmental stressors likely add another layer of cathepsin regulation. Hypoxia is a defining feature of PDAC and profoundly shapes metabolic adaptation, stromal remodeling, and treatment resistance. Direct evidence of HIF-1α binding to cathepsin promoters in bona fide PDAC models remains limited. Studies in non-pancreatic tumor systems have shown that HIF-1α can directly induce CTSB and CTSL transcription [95,96], making this mechanism biologically plausible but not yet firmly established in PDAC. Extracellular acidosis and impaired perfusion may further enhance cathepsin activation by promoting lysosomal instability and facilitating protease activity within acidic extracellular niches.

Inflammatory and cytokine-associated signaling pathways may also contribute to cathepsin regulation within the PDAC TME, although direct mechanistic evidence remains comparatively sparse. NF-κB and STAT3 signaling are central inflammatory pathways in pancreatic cancer and have been implicated in macrophage polarization, stromal activation, and immune suppression. In macrophage systems, IL-4 together with IL-6 or IL-10 induces a cathepsin-secretory phenotype through STAT3/STAT6–IRE1α signaling [97], suggesting that inflammatory cytokines within the PDAC TME may similarly modulate extracellular protease activity. Transcription factors such as Sp1, Sp3, and Ets1 have been implicated in CTSB promoter regulation in other malignancies, but direct PDAC-specific evidence for these transcriptional mechanisms remains limited.

Emerging evidence suggests that epigenetic and post-transcriptional mechanisms may influence cathepsin expression in PDAC. Hypomethylation-associated overexpression of CTSK has been linked to poor prognosis and altered immune infiltration patterns in pancreatic cancer [98], providing one of the few direct examples of epigenetic cathepsin regulation in PDAC. However, compared with the extensive evidence supporting TFEB-dependent lysosomal remodeling, data regarding miRNA-mediated regulation of major PDAC-associated cathepsins remain comparatively sparse and largely correlative.

Importantly, cathepsin biology in PDAC cannot be understood solely from transcript abundance. Cathepsin function is also shaped by lysosomal acidity, zymogen maturation, inhibitor balance, subcellular localization, and extracellular secretion [92,93,94,99]. For example, gemcitabine-induced lysosomal remodeling increases CTSB enzymatic activity rather than simply altering transcript levels [92], while CSTB-dependent stabilization of CTSB activity supports autophagic flux and metabolic adaptation [94]. Altered trafficking is also relevant, as pancreatic cancer stem-like cells show increased surface-associated and secreted CTSB, linking extracellular proteolysis to invasive potential [93]. CTSD provides another example of lysosome-associated adaptation: high CTSD expression correlates with reduced survival in gemcitabine-treated PDAC cohorts, and CTSD silencing partially restores gemcitabine sensitivity in resistant cells, possibly through effects on acid sphingomyelinase activity and lysosomal stress responses [92]. Mechanical features of the TME may further influence lysosomal protease activity, as soft stromal conditions have been reported to promote lysosomal biogenesis, activate cysteine cathepsins, and facilitate YAP1 degradation under acidic conditions [99].

Overall, current evidence supports a model in which cathepsin dysregulation in PDAC reflects coordinated remodeling of the autophagy–lysosome network rather than isolated transcriptional activation of individual proteases. The strongest support is for KRAS–MAPK-, TFEB/MiT-TFE-, therapy-, and lysosomal trafficking–dependent mechanisms. In contrast, hypoxia-, inflammatory-, epigenetic-, miRNA-, and mechanotransduction-associated mechanisms remain biologically plausible but are incompletely validated in PDAC-specific systems. In particular, the broader relevance of stromal-mechanics-dependent cathepsin–YAP1 regulation across heterogeneous human PDAC remains uncertain. Direct promoter-level validation of HIF-1α-, NF-κB-, STAT3-, or Sp1-dependent cathepsin regulation is still limited, and many datasets do not distinguish tumor-cell-derived cathepsin activity from stromal or immune-cell contributions. Future studies integrating spatial transcriptomics, activity-based protease profiling, lysosomal flux analysis, and single-cell approaches will be essential to define the compartment-specific regulation and functional significance of cathepsins in pancreatic cancer progression and therapeutic resistance.

### 3.4. Non-Canonical Release into the Extracellular Space and Microenvironmental Effects

Although typically confined to lysosomes, cathepsins can be redistributed under pathological conditions (reviewed in detail in [100]). In PDAC, the best-supported non-canonical cathepsin function is extracellular and pericellular proteolysis, where secreted or surface-associated cathepsins remodel the matrix-rich tumor microenvironment and support invasive growth [63,80]. Lysosomal membrane permeabilization can also release cathepsins into the cytosol, where they contribute to mitochondrial apoptosis by cleaving the pro-apoptotic protein Bid and degrading anti-apoptotic Bcl-2 family proteins, amplifying caspase-dependent cell death pathways [101]. Nuclear cathepsin functions have also been described in selected systems, although their relevance in PDAC remains less well established [92].

CTSB and CTSL are frequently redirected to pericellular and extracellular sites within the PDAC TME [102,103]. This redistribution appears to reflect adaptive lysosomal trafficking and secretion rather than passive lysosomal leakage, thereby increasing local proteolytic flux. Hypoxia and extracellular acidification are also likely to potentiate extracellular cathepsin activity through lysosomal adaptation and altered trafficking programs. While TFEB-associated lysosomal remodeling is supported in PDAC, direct HIF-1α-dependent regulation of cathepsin expression has been demonstrated more clearly in non-PDAC systems and therefore remains incompletely validated in pancreatic cancer [104]. Although CTSB and CTSL exhibit maximal activity at acidic pH, their enzymatic function is preserved in the PDAC TME (pH ~6.5–6.8) due to local acidification in hypoxic and desmoplastic regions, as well as stabilization through interactions with cell surfaces and ECM components [105]. The resulting pericellular proteolytic niche likely contributes to ECM remodeling and basement membrane disruption, thereby facilitating invasive tumor phenotypes [85,106]. Extracellular CTSB and CTSL degrade key structural ECM proteins, including type I and IV collagens, laminins, and fibronectin, and synergize with matrix metalloproteinases (MMPs) and the urokinase-type plasminogen activator (uPA) system to amplify proteolytic cascades [71,107,108]. Among extracellular cathepsin functions, CTSB/CTSL-mediated ECM remodeling and invasion represent one of the best-supported PDAC-associated mechanisms.

Oncogenic signaling pathways further reinforce this extracellular cathepsin program. In KRAS-driven PDAC, KRAS signaling enhances lysosomal biogenesis and function, expanding the intracellular pool of cathepsins available for secretion. Consistently, DOCK8, a cytoskeletal regulator involved in membrane trafficking and immune signaling, increases lysosomal CTSB activity and promotes tumor cell invasion, linking oncogenic circuitry to enhanced lysosomal proteolysis [106]. AGR2 has also been implicated in regulating CTSB and CTSD expression and activity, supporting metastatic competence. In parallel, Hedgehog signaling can upregulate CTSB expression, further enhancing invasive behavior and stromal–tumor crosstalk [85,109].

Beyond ECM degradation, selected cathepsins may also influence adhesion signaling and integrin-dependent tumor–stroma interactions. For example, CTSZ/X contains an RGD integrin-binding motif and has been linked to integrin-associated invasive behavior in pancreatic tumor models [80,110]. However, much of the strongest mechanistic evidence derives from PanNET or non-PDAC systems, and the significance of CTSZ/X-associated signaling in PDAC remains incompletely resolved. Additional non-canonical release mechanisms also contribute to extracellular cathepsin activity. Lysosomal exocytosis can deposit active lysosomal peptidases directly into the pericellular space, further promoting ECM remodeling and tumor invasion [34]. Moreover, CTSC, a lysosomal peptidase that activates neutrophil serine proteases such as neutrophil elastase, proteinase 3, and CTSG during neutrophil maturation, links lysosomal peptidase activity to inflammatory and immune responses within the TME [111].

Taken together, non-canonical trafficking and secretion pathways extend cathepsin activity beyond lysosomes and integrate extracellular matrix remodeling, invasion, inflammatory protease networks, and lysosome-dependent stress adaptation. However, the strength of evidence varies substantially across proposed mechanisms. Extracellular CTSB/CTSL-associated matrix remodeling is strongly supported in PDAC, whereas adhesion signaling, inflammatory amplification, and immune-regulatory functions remain more context-dependent and incompletely validated.

### 3.5. Clinical Associations and Compartmental Expression

Multiple members of the cathepsin family are dysregulated in PDAC and have been associated with clinical outcomes [85,106,112]. Transcriptomic, proteomic, and immunohistochemical studies consistently link altered cathepsin expression with patient survival, tumor progression, and metastatic potential, supporting their functional contribution to PDAC pathobiology and their potential prognostic value [82,102,106,113]. Importantly, cathepsin expression and activity vary substantially across malignant epithelial, stromal, and immune compartments, indicating that cathepsin dysregulation in PDAC reflects a broader compartment-specific lysosomal adaptation rather than a purely tumor-cell-intrinsic phenomenon. Consequently, interpretation of cathepsin expression requires consideration of cellular source, enzymatic activity state, and microenvironmental context. Among PDAC-associated cathepsins, CTSB, CTSL, and CTSD are most consistently linked to aggressive tumor behavior and adverse clinical outcomes. Elevated CTSB and CTSL expression correlates with invasive growth, metastatic dissemination, and reduced survival [69,114]. Mechanistically, these proteases contribute to ECM remodeling, basement membrane degradation, and stromal remodeling, thereby facilitating tumor invasion and dissemination [71,108]. Experimental studies further suggest that lysosomal permeabilization and cytosolic cathepsin release may support stress adaptation and inflammatory signaling under therapeutic pressure [115], although the relative importance of these intracellular mechanisms in human PDAC remains incompletely resolved. CTSL has also been associated with neural invasion and perineural dissemination, both characteristic features of aggressive PDAC biology [102]. Immunohistochemical studies show that CTSL immunoreactivity is often prominent in the desmoplastic stroma; however, epithelial expression appears to have greater prognostic significance. Increased tumor-cell-associated CTSL expression correlates with lymphatic invasion, advanced tumor stage, early postoperative recurrence, and reduced overall survival [113]. These findings indicate that tumor cell–derived CTSL, rather than stromal expression alone, more accurately reflects aggressive tumor behavior and may serve as a clinically meaningful prognostic marker. These observations further support the importance of compartment-resolved biomarker assessment when evaluating lysosome-targeted or cathepsin-responsive therapeutic strategies [116]. Cathepsin V (CTSV; UniProt ID: O60911), also known as cathepsin L2 or cathepsin U, is a close paralog of cathepsin L that shares elastolytic and collagenolytic properties and has been implicated in invasive behavior in several epithelial malignancies [117]. Transcriptomic analyses indicate detectable CTSV expression in PDAC; however, direct associations with prognosis, chemoresistance, or metastasis remain limited. Given its structural similarity to CTSL, CTSV may participate in ECM remodeling at the tumor–stroma interface, although compartment-resolved evidence in pancreatic cancer is still emerging. CTSS appears to occupy a more complex position at the interface between stromal remodeling and immune regulation. In addition to extracellular matrix degradation and invasive growth [118], CTSS participates in MHC class II antigen-processing pathways and macrophage-associated inflammatory responses. Because CTSS contributes to antigen processing, altered CTSS activity could plausibly influence immune regulation within the PDAC TME. However, direct evidence demonstrating that CTSS-driven antigen-processing defects promote immune evasion in human PDAC remains limited. Therefore, CTSS should currently be regarded as a promising but incompletely validated immunomodulatory target rather than an established driver of immune escape [119,120]. Bioinformatic analyses have further identified CTSB as a central “hub” gene within PDAC-associated regulatory networks [121]. Functional studies support its role in sustaining invasive growth, lysosomal stress adaptation, and therapy resistance in PDAC models [114,120]. Because CTSB activity is strongly enriched within invasive tumor margins and stem-like tumor cell populations, CTSB has also emerged as a promising candidate for activity-based imaging probes and protease-activatable therapeutic delivery systems designed to selectively target proteolytically active PDAC lesions. Increased CTSB expression correlates with adverse pathological features, poorer surgical outcomes, and enrichment within pancreatic cancer stem-like subpopulations [104]. Importantly, several cathepsins appear to reflect stromal or immune-cell programs rather than purely malignant epithelial biology. CTSC is a lysosomal cysteine peptidase best known for activating neutrophil serine peptidases, including neutrophil elastase, proteinase 3, and CTSG, during neutrophil maturation. Consequently, CTSC expression in tumors often reflects myeloid or neutrophil infiltration rather than tumor cell–intrinsic activity [122]. Thus, associations between CTSC expression and PDAC prognosis likely reflect inflammatory myeloid activity within the tumor microenvironment rather than direct tumor-cell functions [123,124]. CTSH is detectable in PDAC across malignant epithelial and stromal compartments and has been identified in endoscopic ultrasound–guided fine needle aspiration (EUS-FNA) cytology material from pancreatic lesions. However, current datasets have not established a consistent relationship between CTSH expression levels and disease stage or clinical outcome [81].

Among structurally distinct cathepsins, CTSZ/X has strict carboxypeptidase activity and contains a unique exposed RGD integrin-binding motif within its propeptide region [125]. Unlike classical matrix-degrading cathepsins, CTSZ/X often promotes tumor progression through integrin signaling rather than proteolysis alone. In pancreatic neuroendocrine tumor (PanNET) models, CTSZ/X is produced by both tumor cells and TAMs, but its functional contributions are compartment-specific. Tumor cell–derived CTSZ/X enhances proliferation and invasion via RGD-dependent integrin engagement and activation of FAK–Src signaling, whereas macrophage-derived CTSZ/X mainly promotes invasion without directly affecting tumor cell proliferation [80]. Although these mechanistic insights derive primarily from PanNET models, the integrin–FAK–Src signaling axis is also highly active in PDAC, and CTSZ/X regulation through S100PBP has been demonstrated in pancreatic cancer cells, suggesting that similar integrin-mediated programs may operate in PDAC [80]. Given the importance of FAK–Src signaling in PDAC invasion and stromal communication, CTSZ/X-associated integrin signaling may represent a therapeutically relevant vulnerability that warrants further investigation in compartment-resolved PDAC models. In contrast, CTSW exhibits fundamentally different biology, being expressed predominantly in cytotoxic lymphocytes such as CD8^+^ T cells and natural killer (NK) cells [126]. Transcriptomic analyses of TCGA datasets show that reduced CTSW expression correlates with decreased overall survival in PDAC patients [82]. Because CTSW expression is restricted to cytotoxic immune cells, reduced levels likely reflect diminished immune infiltration rather than tumor cell–intrinsic peptidase activity. Accordingly, CTSW has been incorporated into machine-learning–based survival models as an immune-context biomarker of cytotoxic antitumor activity [82]. Reduced CTSW expression may therefore identify PDAC tumors with impaired cytotoxic immune infiltration and potentially lower responsiveness to immunotherapeutic approaches.

CTSK is a highly collagenolytic cysteine peptidase best known for its role in bone remodeling but is also conceptually relevant to PDAC due to the dense collagen-rich desmoplastic stroma. Transcriptomic studies suggest that CTSK expression signatures correlate with stromal or immune features and clinical outcomes in selected pancreatic cancer cohorts [98]. However, mechanistic studies defining its cellular source and functional role in PDAC remain limited. Cathepsin F (CTSF; UniProt ID: Q9UBX1) is another lysosomal cysteine peptidase for which PDAC-specific evidence remains sparse. Transcriptomic datasets confirm CTSF expression in pancreatic tumors [99,127], but consistent associations with patient survival, metastatic progression, or therapy response have not yet been established. Among aspartic cathepsins, CTSD is frequently upregulated in PDAC tumor epithelium. Elevated CTSD expression correlates with reduced overall survival and adverse clinicopathologic features [120]. Integrative translational analyses further indicate that CTSD contributes to reduced responsiveness to gemcitabine, linking lysosomal peptidase activity to chemotherapy resistance [92]. Thus, CTSD is best interpreted as a tumor-intrinsic peptidase associated with aggressive disease behavior and therapeutic tolerance [92]. Mechanistically, CTSD may contribute to chemoresistance through lysosome-mediated stress adaptation and activation of survival signaling pathways under metabolic and therapeutic stress conditions. These observations support further exploration of CTSD-associated lysosomal signaling as a potential therapeutic target in gemcitabine-resistant PDAC. In contrast, CTSE displays one of the most disease-selective expression patterns among pancreatic cathepsins. CTSE is markedly upregulated in pancreatic intraepithelial neoplasia (PanIN) lesions and invasive PDAC compared with benign pancreatic tissue [87,128]. Elevated CTSE levels have also been detected in pancreatic juice from affected patients [123]. Unlike CTSD, CTSE has not been consistently linked to metastasis or therapy resistance; rather, its strongest clinical association lies in lesion-enriched epithelial expression and diagnostic specificity, making CTSE a promising biomarker for early detection of PDAC [87,128]. Because CTSE expression appears relatively enriched in pancreatic precursor lesions and malignant epithelial compartments, CTSE-responsive molecular imaging probes and theranostic approaches may offer additional opportunities for early disease detection and lesion-specific targeting [86]. The prognostic significance of altered cathepsin expression in PDAC likely relates to the distinct biological functions of individual cathepsins within the TME. Increased CTSB and CTSL expression is associated with enhanced ECM degradation, basement membrane remodeling, and activation of invasive signaling pathways, processes that may contribute to lymphatic dissemination, tumor recurrence, and shorter patient survival. In addition to its role in pericellular proteolysis, CTSB has also been implicated in maintaining stem-like tumor cell properties and adaptation to lysosomal stress, which may further support tumor aggressiveness and resistance to therapy.

Elevated CTSD expression has also been linked to poor clinical outcomes and reduced sensitivity to gemcitabine, consistent with its proposed role in stress adaptation and survival signaling during chemotherapy [92]. The prognostic relevance of CTSS appears to extend beyond ECM remodeling, as this protease may also influence immune regulation through effects on antigen processing and macrophage-associated inflammatory responses [119]. In contrast, reduced CTSW expression most likely reflects decreased infiltration of cytotoxic immune cells rather than tumor-derived proteolytic activity, suggesting an association with impaired antitumor immunity [82].

These functional differences may also have therapeutic relevance. CTSB, CTSL, CTSD, and CTSS have been proposed as potential targets for selective cathepsin inhibition, lysosome-directed therapies, or cathepsin-responsive drug delivery systems. In contrast, CTSW may be more valuable as an indicator of immune context and patient stratification. Taken together, current evidence suggests that cathepsin dysregulation in PDAC reflects a broader adaptive lysosomal state integrating oncogenic KRAS signaling, stromal remodeling, extracellular matrix turnover, metabolic stress adaptation, and immune-context-dependent proteolysis rather than isolated enzyme-specific abnormalities. The strongest PDAC-specific evidence supports CTSB/CTSL-associated invasion, CTSD-associated therapy resistance, CTSE-associated diagnostic enrichment, and TFEB/KRAS-linked lysosomal remodeling. In contrast, several proposed immune-regulatory, mechanotransduction-associated, and stromal-signaling functions remain context-dependent or incompletely validated. Future studies incorporating spatially resolved profiling, activity-based protease mapping, and functional perturbation models will be essential for distinguishing tumor-cell-derived, stromal, and immune-cell-associated cathepsin programs in human PDAC.

**Table 1:** Functional and Clinical Classification of Cathepsins in PDAC.

Cathepsin	Major Cellular Source in PDAC	Main Biological Function	Prognostic Significance	Therapeutic Relevance	Ref.
CTSB	Tumor cells, invasive margins, stromal/immune compartments	ECM degradation, invasion, lysosomal stress adaptation, stem-like phenotype	High expression/activity linked to aggressive disease and poorer outcome	Potential inhibitor target; strong candidate for activity-based imaging and protease-activatable drug delivery	[69,114]
CTSL	Tumor epithelium and stroma	ECM remodeling, invasion, possible perineural/lymphatic dissemination	High epithelial expression associated with recurrence and reduced survival	Potential target; often more realistic in combination or activity-based delivery strategies	[102]
CTSS	APCs, macrophages, stromal/tumor-associated compartments	MHC-II processing, macrophage-associated inflammation, ECM remodeling	Prognostic role context-dependent	Immunomodulatory target, but broad inhibition may impair antigen presentation	[119]
CTSD	Tumor epithelium	Lysosomal stress adaptation, survival signaling, gemcitabine resistance	High expression linked to poor survival and reduced gemcitabine response	Candidate biomarker and possible combination target in chemoresistant PDAC	[120]
CTSE	PanIN and PDAC epithelial lesions	Disease-enriched lysosomal/aspartic protease activity	Diagnostic enrichment rather than established prognostic marker	Strong imaging/theranostic biomarker; less clearly a direct therapeutic target	[123]
CTSZ/X	Tumor cells, macrophages; stronger evidence from PanNET and pancreatic cancer models	Integrin-associated adhesion and invasion	Prognostic role in PDAC incompletely defined	Investigational target; requires PDAC-specific validation	[80]
CTSC	Myeloid/neutrophil-associated compartments	Activation of neutrophil serine proteases, inflammatory proteolysis	May reflect inflammatory myeloid infiltration	More likely immune context biomarker or inflammatory modulation target	[122]
CTSW	CD8^+^ T cells and NK cells	Cytotoxic immune-cell-associated protease	Low expression associated with poor survival, likely reflecting reduced cytotoxic infiltration	Immune-context biomarker, not a tumor-cell drug target	[82]
CTSK	Stromal/immune-associated signatures; cellular source unclear	Collagenolysis; possible stromal remodeling	Bioinformatic association with prognosis/immune infiltration	Requires mechanistic validation	[98]
CTSF	Detected in pancreatic tumors; functional role unclear	Unknown/limited PDAC-specific evidence	Possible association with susceptibility in genetic analyses	Biomarker hypothesis only; not currently a validated therapeutic target	[99,127]

## 4. Lysosome–Cathepsin Axis in Anti-Tumor Immunity in Pancreatic Cancer

### 4.1. Antigen Presentation: Role of Cathepsins in MHC Class II Loading in Dendritic Cells and Macrophages

**Figure 2:**
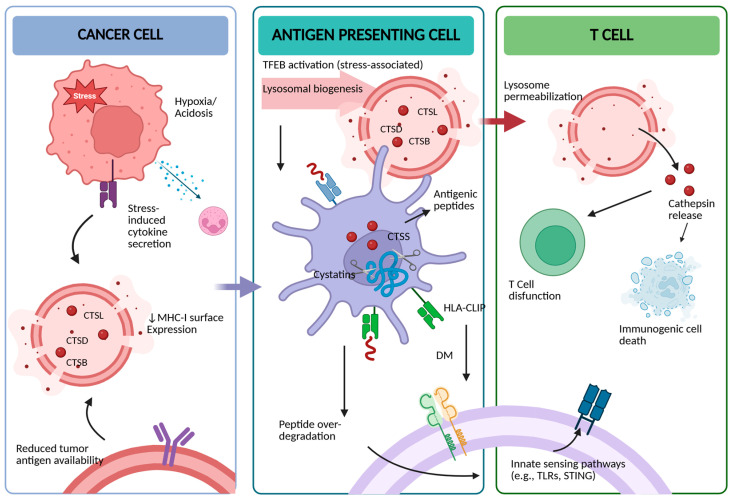
Regulation of Antitumor Immunity by the Lysosome–Cathepsin Axis (Tumor–APC–T Cell Interface). The lysosome–cathepsin axis plays a context-dependent role in regulating antitumor immunity in the TME. In cancer cells, stress-associated lysosomal activity and cathepsin function may alter antigen processing and reduce tumor antigen availability. In APCs, lysosomal peptidases, particularly cathepsin S (CTSS), regulate MHC class II antigen processing, while excessive proteolysis may limit peptide presentation. In T cells, lysosomal membrane permeabilization and cathepsin release can impair cellular function and survival. Collectively, these processes contribute to reduced antigen presentation and T cell dysfunction. Abbreviations: Major Histocompatibility Complex (MHC); Transcription Factor EB (TFEB); TLR9 (Toll-like receptor 9); STING (stimulator of interferon genes); CLIP, class II–associated invariant chain peptide; DM, HLA-DM, a molecule that facilitates peptide loading onto MHC class II by removing CLIP. (Created with https://BioRender.com).

A central immune function of lysosomal cathepsins is processing internalized antigens for presentation (see Figure 2). In APCs, such as dendritic cells, macrophages, and B cells, endolysosomal cathepsins generate peptide ligands from internalized proteins and shape the repertoire available for loading onto MHC-II molecules. A key step in this pathway is the proteolytic processing of the invariant chain (Ii), which occupies the MHC-II peptide-binding groove during biosynthesis. Among lysosomal peptidases, CTSS is the principal enzyme driving invariant-chain cleavage in dendritic cells, macrophages, and B cells, enabling removal of Ii-derived intermediates, formation of CLIP-containing complexes, and subsequent peptide exchange for stable MHC-II loading and CD4^+^ T-cell presentation. CTSL can also contribute to this pathway, although its role is more context- and cell-type-dependent. Along with endosomal acidification, cathepsin activity determines whether antigenic epitopes are preserved for presentation or over-degraded before loading, making lysosomal proteolysis a key checkpoint in adaptive immune priming [20,129]. Recent work continues to support CTSS as a critical regulator of MHC-II antigen presentation and CD4^+^ T-cell activation.

### 4.2. Implications for T-Cell Priming in PDAC

In PDAC, lysosome-dependent antigen handling is altered in ways that weaken both tumor cell recognition and APC-driven T-cell priming. Under normal physiological conditions, MHC-I molecules present endogenously derived peptides to CD8^+^ T cells through a tightly regulated intracellular pathway. Intracellular proteins are first processed by the proteasome into short peptide fragments, which are then transported into the endoplasmic reticulum via the TAP1/TAP2 complex. Within the ER, these peptides are loaded onto newly synthesized MHC-I molecules in association with β2-microglobulin and the peptide-loading machinery before being transported to the plasma membrane for immune surveillance. Although lysosomes are not directly responsible for peptide generation in the classical MHC-I pathway, they contribute to MHC-I turnover and intracellular quality control by degrading internalized or improperly trafficked complexes [130].

Surface MHC-I is frequently reduced through autophagy–lysosome-dependent trafficking and degradation, limiting CD8^+^ T-cell recognition and reinforcing the immunologically “cold” phenotype of PDAC [130]. Instead of maintaining stable membrane-associated MHC-I expression, PDAC cells can aberrantly reroute MHC-I complexes to autophagosomal and lysosomal compartments, leading to their degradation and reduced antigen presentation at the cell surface. Notably, this reduction in MHC-I expression often occurs without recurrent genetic defects in core antigen-presentation machinery, indicating that post-translational lysosomal trafficking mechanisms substantially contribute to immune evasion in PDAC. Mechanistically, MHC-I is selectively diverted from the plasma membrane into autophagosomes and lysosomes via an NBR1-dependent pathway, where it is degraded; inhibition of autophagy or lysosomal function restores surface MHC-I and enhances CD8^+^ T-cell–mediated tumor control in PDAC models. Recent work further suggests that selective cargo-capture machinery involving NBR1 and the ER-phagy receptor TEX264 may facilitate this routing, although this extension is currently based on a 2024 preprint rather than a peer-reviewed study [60,131,132]. These findings identify the autophagy–lysosome system as a mechanistic link between metabolic stress adaptation and immune evasion in PDAC. Consistent with this, PDAC often shows reduced MHC-I expression without recurrent inactivating mutations in core MHC-I genes, supporting the idea that defective antigen display is driven, at least in part, by post-translational lysosomal routing rather than irreversible genetic loss.

At the level of APCs, altered CTSS-dependent endolysosomal proteolysis in tumor-associated macrophages or dendritic cells could plausibly distort invariant-chain processing, peptide trimming, and the quality of MHC-II presentation, thereby weakening CD4^+^ T-cell priming. In parallel, PIKfyve-dependent phagosome-to-lysosome maturation helps establish the endolysosomal environment required for efficient CTSS-mediated antigen processing and MHC-II presentation; inhibition of this pathway reduces CD4^+^ T-cell activation [133]. Together, these lysosome-dependent mechanisms can simultaneously limit tumor-cell visibility to CD8^+^ T cells and compromise APC-mediated T-cell priming, helping explain the weak adaptive immune responses typical of PDAC [60,130,134].

Beyond antigen presentation, lysosomal cathepsins also influence inflammatory signaling by regulating endolysosomal maturation, receptor turnover, and cytokine-processing pathways. Through proteolytic control of ligands, receptors, and signaling intermediates, they can either amplify or restrain inflammatory outputs, further shaping the immune tone of the PDAC TME [135].

### 4.3. Lysosomal Cathepsins in Stromal Remodeling and Immune Exclusion

TAMs in PDAC are often skewed toward an immunosuppressive, tissue-remodeling phenotype that promotes fibrosis, invasion, and immune evasion. In this context, lysosomal cysteine cathepsins contribute not only to ECM turnover but also to macrophage metabolic programming. Pharmacologic inhibition of CTSB, CTSL, and CTSS with GB111-NH2 in murine and human tumor-infiltrating macrophages reduced M2-like features, induced a shift toward a more pro-inflammatory phenotype, and altered lysosome-linked metabolic pathways, including ATP production and lipid mediator profiles. These findings support the view that cysteine cathepsins are active regulators of TAM polarization rather than passive markers of macrophage state, with direct implications for stromal remodeling in PDAC [136].

In parallel, stromal mechanical cues intersect with lysosomal proteolysis to influence tumor-cell behavior. Recent work shows that in compliant stromal environments, the autophagic–lysosomal axis promotes cathepsin-dependent degradation of YAP1, thereby constraining proliferative outgrowth. In PDAC tissues, CTSL and YAP1 show an inverse relationship, supporting a model in which reduced lysosomal cathepsin activity permits YAP1 persistence and favors tumor progression. This is particularly relevant because stromal stiffening, fibrosis, and altered lysosomal function are tightly linked in PDAC [99].

Together, these observations suggest that lysosomal peptidase activity in both myeloid and tumor compartments contributes to desmoplastic remodeling, sustains stromal–tumor crosstalk, and reinforces immune exclusion in PDAC. In this context, the lysosome–cathepsin axis can be viewed as a determinant of immune accessibility, acting through coordinated ECM remodeling, mechanotransduction, and modulation of macrophage polarization.

### 4.4. Lysosomal Membrane Permeabilization and Cathepsin-Mediated Cell Death

Beyond extracellular proteolysis, cathepsins also interact with autophagy and stress-adaptation pathways in PDAC. Under homeostatic conditions, CTSB regulates lysosomal biogenesis and autophagy by cleaving the lysosomal Ca^2+^ channel MCOLN1/TRPML1. This cleavage limits activation of the transcription factor TFEB, thereby restraining lysosomal biogenesis and the expression of autophagy-related genes. Consistent with this regulatory role, combined deficiency of CTSB and the aspartic peptidase CTSD in pancreatic tissue disrupts autophagic homeostasis, supporting a broader role for lysosomal peptidases in maintaining degradative flux [137,138]. In PDAC cells, sustained CTSB activity supports autophagic cargo degradation and generates metabolites that can fuel central metabolic pathways, including glycolysis. Autophagy in PDAC critically depends on ATG5, which is required for LC3-II–positive autophagosome formation and maintenance of autophagic flux [77]. Genetic studies in PDAC models driven by oncogenic signaling and tumor suppressor loss demonstrate a context-dependent role for autophagy: deletion of ATG5 can enhance tumor initiation but impair later tumor progression. Complete ATG5 loss blocks tumorigenesis, whereas monoallelic ATG5 loss promotes tumor development and metastasis [77]. In ATG5^+^/^−^; KRAS-driven PDAC models, partial impairment of autophagy increases extracellular activity of CTSL and CTSD, peptidases associated with ECM degradation and tumor invasion. This alteration links lysosomal homeostasis and cathepsin activity to cytokine secretion and immune modulation within the TME. In the same model, cytokine profiles shift toward signals that favor pro-tumorigenic macrophage polarization, with increased infiltration of M2-like macrophages observed in both primary and metastatic lesions. Consistently, macrophage depletion in these models reduces metastatic burden, supporting a role for lysosome-dependent peptidase activity in shaping stromal and immune dynamics [77]. Under conditions of tumor stress or lysosomal dysfunction, lysosomal membrane permeabilization (LMP) can occur. This process releases cathepsins from the lysosomal lumen into the cytosol, where they shift from degradative roles within lysosomes to cytosolic effectors capable of activating apoptotic and inflammatory signaling pathways. Through these mechanisms, lysosomal destabilization links metabolic stress, autophagy disruption, and cathepsin activity to cell death and inflammatory responses in PDAC [67].

Release of lysosomal cathepsins following LMP can trigger apoptosis through both caspase-dependent and caspase-independent mechanisms. In PDAC models, several therapeutic agents exploit this pathway. In BxPC-3 pancreatic cancer cells, the proteasome inhibitor PS-341 (bortezomib) induces apoptosis by promoting reactive oxygen species (ROS)–dependent lysosomal permeabilization and cytosolic release of CTSB. Cytosolic CTSB subsequently activates caspase-2, linking lysosomal damage to mitochondrial depolarization and downstream apoptotic signaling [139]. Similarly, Bobel-24, a cyclooxygenase/5-lipoxygenase (COX/5-LOX) inhibitor, induces lysosomal permeabilization and CTSB release, leading to mitochondrial depolarization, ROS accumulation, and apoptosis-inducing factor (AIF) translocation. In contrast to PS-341, this pathway proceeds largely independently of caspase activation, demonstrating that CTSB-mediated mitochondrial damage can also drive caspase-independent cell death in apoptosis-resistant pancreatic cancer cells such as NP9 [140].

Beyond apoptosis, emerging evidence suggests that CTSB may also participate in ferroptosis-related pathways. Iron-dependent lysosomal disruption can promote CTSB release and nuclear translocation, leading to DNA damage and activation of STING1 signaling [141]. In pancreatic cancer cells, nuclear CTSB has been proposed to facilitate autophagy-dependent ferroptosis; however, the extent to which this mechanism contributes to PDAC progression or therapeutic response in vivo remains incompletely established [142].

## 5. Therapeutic Targeting of the Lysosome-Cathepsin Axis

### 5.1. Lysosome-Targeting Drugs

Lysosome-targeted therapy has emerged as a promising strategy in PDAC by exploiting the tumor’s reliance on elevated autophagy and lysosome-mediated metabolic recycling (see Table 2). Therapeutic approaches have primarily focused on inhibiting late-stage autophagy and lysosomal function, most commonly using chloroquine (CQ) or hydroxychloroquine (HCQ), which interfere with autophagosome–lysosome fusion and blunt a major stress-adaptation pathway in PDAC cells [143,144]. These strategies represent early autophagy-targeting approaches that have evolved into broader efforts to exploit tumor-specific lysosomal dependency. Preclinical studies show that gemcitabine treatment induces autophagy and lysosomal activity through ERK–TFEB signaling, reflecting a therapy-induced lysosomal “rescue” response in PDAC cells [78]. Conversely, CQ sensitizes PDAC cells to gemcitabine by increasing oxidative stress and promoting lysosomal membrane permeability, leading to protease release and apoptosis [145].

Activation of TFEB and induction of lysosomal and autophagy-related gene expression are strongly associated with PDAC growth and metabolic fitness [146,147]. Accordingly, next-generation lysosomal inhibitors have been developed to target this dependency. The palmitoyl-protein thioesterase 1 (PPT1) inhibitor DC661 induces lysosomal membrane permeabilization, lipid peroxidation, and immunogenic cell death, although resistance mechanisms involving lipid metabolic rewiring, particularly glycosphingolipid metabolism, have also been described [141,148]. In addition, PIKfyve, a kinase regulating lysosomal lipid homeostasis and membrane dynamics, has emerged as a therapeutic vulnerability distinct from classical lysosomal acidification blockade [149].

Clinical evaluation of HCQ in combination regimens has shown biological activity but limited efficacy as a single agent, underscoring the need for rational therapeutic combinations [150]. Adaptive resistance can occur through TFEB-driven lysosomal biogenesis and drug sequestration, particularly following MAPK pathway inhibition, reinforcing the concept of PDAC “lysosomal addiction” [151]. In metastatic PDAC, the randomized phase II trial by Karasic et al. demonstrated that adding HCQ to gemcitabine/nab-paclitaxel improved response rates and CA19-9 responses, but failed to significantly improve overall survival, while also increasing treatment-related toxicities [152]. Similarly, Wolpin et al. reported pharmacodynamic evidence of autophagy inhibition in metastatic PDAC patients treated with HCQ; however, clinical benefit remained limited, suggesting that autophagy blockade alone is insufficient to overcome therapeutic resistance in PDAC [153,154]. More recently, an open-label phase II study evaluating the ERK inhibitor LY3214996 in combination with HCQ in metastatic PDAC showed minimal clinical activity, despite strong preclinical rationale supporting dual ERK/autophagy inhibition [155]. These findings suggest that compensatory metabolic rewiring and incomplete suppression of autophagy may continue to limit the efficacy of lysosome-targeting strategies, even in rationally designed combination regimens. Emerging translational analyses suggest that the modest efficacy of HCQ/CQ-based strategies may be explained by inadequate intratumoral drug accumulation and adaptive lysosomal compensation. CQ and HCQ are weak-base lysosomotropic agents whose distribution is strongly influenced by extracellular pH gradients and stromal architecture. In PDAC, the dense desmoplastic stroma, elevated interstitial fluid pressure, poor vascularization, and heterogeneous perfusion substantially impair effective drug penetration into tumor tissue. Moreover, acidic and hypoxic regions within the PDAC TME may alter lysosomal drug-trapping dynamics, reducing effective target inhibition in cancer cells [21,156,157,158].

TFEB-driven compensatory lysosomal biogenesis has emerged as an additional key resistance mechanism limiting durable responses to lysosomal inhibition. Pharmacologic autophagy blockade can paradoxically activate TFEB-dependent transcriptional programs that restore lysosomal capacity and metabolic adaptation, enabling PDAC cell survival despite continued HCQ exposure. This adaptive response appears particularly relevant in KRAS/MAPK-driven tumors, where ERK inhibition and lysosomal stress converge on TFEB activation and enhanced lysosomal recycling pathways [151,159,160]. These findings provide a mechanistic explanation for the disconnect between strong preclinical activity and modest clinical efficacy observed in HCQ-based trials. In contrast, in the neoadjuvant setting, HCQ-based combinations improved pathological responses and demonstrated pharmacodynamic evidence of autophagy inhibition and immune activation, supporting biomarker-guided patient selection [153]. The randomized phase II preoperative trial by Zeh et al. showed significantly improved histopathologic responses in patients receiving HCQ combined with gemcitabine and nab-paclitaxel, although this did not translate into a statistically significant survival advantage [153,161]. These results suggest that lysosomal inhibition may be more effective in earlier-stage disease or in biologically selected patient subsets. Recent studies further indicate that HCQ-based combinations may exert broader effects on the PDAC TME beyond autophagy inhibition alone. Preclinical and translational analyses of paricalcitol/HCQ-based regimens demonstrated reduced CAF activity, ECM remodeling, and enhanced immune-cell activation, supporting the concept that stromal modulation may improve therapeutic responsiveness in PDAC [162,163]. Beyond CQ derivatives, therapeutic strategies that directly induce lysosomal membrane permeabilization have also been explored. For example, siramesine-like compounds can selectively target pancreatic cancer stem-like cells in patient-derived xenograft models, suggesting an alternative approach to exploiting lysosomal vulnerability in PDAC [156].

Despite encouraging preclinical results, the overall clinical benefit of lysosomal inhibitors in PDAC remains modest. Current evidence suggests that this limitation reflects both tumor-intrinsic adaptive resistance mechanisms and major pharmacologic barriers imposed by the PDAC TME. Consequently, future therapeutic strategies will likely require combination approaches integrating lysosomal inhibition with stromal remodeling, vascular normalization, MAPK pathway blockade, or immunotherapy to achieve sufficient intratumoral drug delivery and prevent compensatory lysosomal reprogramming [21,52,157,160]. Ongoing clinical trials are investigating multi-target approaches that combine HCQ with stromal-modulating agents such as paricalcitol and gemcitabine/nab-paclitaxel in advanced PDAC, reflecting continued efforts to overcome the pharmacologic and microenvironmental limitations of earlier HCQ-based strategies [164].

Collectively, these studies support the concept that PDAC exhibits a functional dependence on lysosomal pathways; however, effective therapeutic exploitation will likely require combination strategies to prevent compensatory lysosomal biogenesis and metabolic adaptation.

**Table 2:** Translational lessons from representative lysosome–cathepsin-targeting strategies in PDAC.

Approach/Representative Refs.	Experimental or Clinical Design	Main Readouts	Advantages	Limitations/Translational Lesson	Ref.
HCQ + gemcitabine/nab-paclitaxel	Metastatic or neoadjuvant phase II PDAC trials	Response rate, CA19-9, histopathologic response, survival, pharmacodynamic autophagy markers	Clinically feasible; tests lysosomal inhibition with standard chemotherapy; tissue-based readouts possible in the neoadjuvant setting	Limited survival benefit; HCQ is nonspecific and weak; intratumoral lysosomal inhibition may be incomplete; poor stromal penetration and TFEB-driven compensation likely limit efficacy	[152]
ERK inhibition + HCQ	Mechanism-driven phase II trial based on MAPK/autophagy crosstalk	Clinical response, disease control, survival	Rationally targets KRAS/MAPK-driven autophagy adaptation	Minimal activity suggests HCQ may not fully suppress therapy-induced lysosomal adaptation; metabolic rewiring may bypass dual blockade	[155]
Direct cathepsin inhibition	Preclinical cell and mouse models using CTSB/CTSL/CTSS or broad cathepsin inhibitors	Viability, invasion, tumor burden, protease activity	Demonstrates the pathogenic role of cathepsin activity in invasion and tumor growth	Often simplified models; limited stromal/immune fidelity; protease redundancy; possible impairment of normal immune proteolysis	[159]
Cathepsin-responsive prodrugs/nanoparticles	Preclinical drug delivery and imaging systems using CTSB/CTSS/CTSE-cleavable linkers	Drug release, fluorescence activation, tumor growth, biodistribution	Exploits tumor protease activity instead of blocking it; improved spatial selectivity	Heterogeneous cathepsin activity; stromal penetration barriers; limited clinical validation; requires pharmacodynamic confirmation of intratumoral cleavage	[165,166]
CTSE-activatable imaging/prodrugs	Preclinical imaging of precursor and invasive pancreatic lesions	Lesion detection, probe activation, imaging contrast	Strong PDAC-enriched expression; useful for diagnostics/theranostics	More suitable as biomarker/imaging target than general therapeutic target; clinical translation remains limited	[167]

### 5.2. Cathepsin Inhibitors

**Figure 3:**
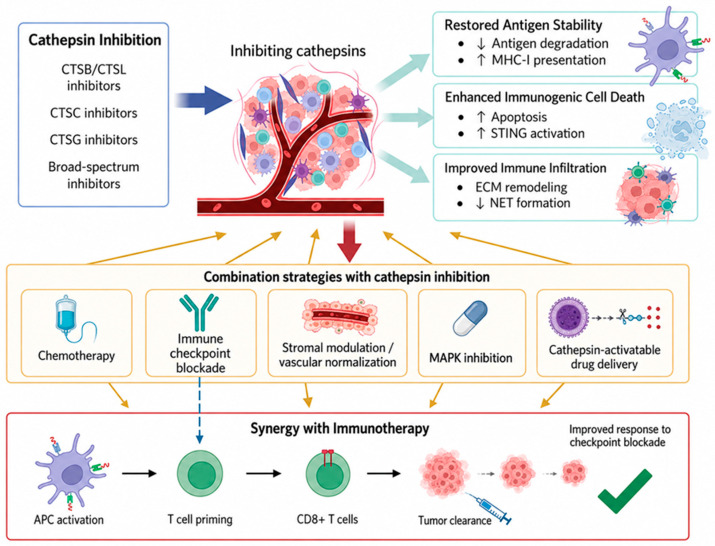
Targeting Cathepsins to Restore Immunogenic Cell Death and Enhance Immunotherapy in PDAC. Pharmacological inhibition of cathepsins is a promising strategy to restore antitumor immunity and improve the efficacy of immunotherapy in PDAC. Inhibition of selected cathepsins may stabilize tumor-derived antigens in some contexts, but broad cathepsin inhibition could also impair antigen processing in professional APCs; therefore, immunological consequences are likely compartment- and treatment-context-dependent. This may contribute to the induction of immunogenic cell death (ICD), characterized by increased apoptotic signaling and activation of innate immune pathways, including STING. Additionally, cathepsin inhibition may modulate ECM-associated barriers and influence NET formation, thereby facilitating immune cell infiltration into the TME. Collectively, these mechanisms are proposed to promote antigen presentation, support APC activation and T-cell priming, enhance CD8^+^ T-cell–mediated tumor clearance, and improve responses to immune checkpoint blockade. Clinically, such approaches are unlikely to be effective as stand-alone strategies. More realistic therapeutic applications include biomarker-guided combinations with chemotherapy, immune checkpoint blockade, stromal remodeling or vascular normalization strategies, MAPK pathway inhibition, and cathepsin-activatable drug delivery systems. These combinations aim to overcome compensatory lysosomal adaptation, improve drug penetration, and preserve or enhance antitumor immune priming (Created with https://BioRender.com).

Direct pharmacologic inhibition of cysteine cathepsins (see Figure 3) offers a complementary strategy to lysosome-targeting therapies by blocking the proteolytic activity of the lysosomal compartment rather than globally disrupting lysosomal pH or trafficking. In PDAC, cathepsin inhibition has been investigated both as a tumor-intrinsic strategy, limiting invasion, proliferation, and stress adaptation, and as a microenvironmental approach that modulates stromal and immune-cell proteolysis.

CTSB, which is frequently upregulated in PDAC, contributes to ECM degradation, invasion, and tumor progression. Preclinical studies using the CTSB-selective inhibitor CA-074Me demonstrated antitumor activity in pancreatic cancer models, including reductions in tumor growth comparable to gemcitabine in certain contexts, providing early proof-of-concept for pharmacologic CTSB inhibition [159,161]. Other cathepsins also contribute to PDAC progression. Co-overexpression of CTSL and CTSS has been linked to metastatic behavior, invasion, and immune modulation. Dual inhibition of CTSL and CTSS using ASPER-29 reduced metastatic traits in vitro [165], while newly developed CTSS inhibitors demonstrated antiproliferative activity in BxPC-3 and Capan-1 pancreatic cancer cells [168]. Broad-spectrum cathepsin inhibition has also shown proof-of-concept activity. The reversible multi-cathepsin inhibitor VBY-825 reduced tumor burden in pancreatic cancer models [166]. Natural compounds can also target lysosomal protease pathways. For example, matrine, an alkaloid derived from Sophora flavescens, suppresses pancreatic cancer growth by disrupting autophagy-dependent energy metabolism. Matrine inhibits lysosomal protease activity by preventing maturation of CTSB and CTSD, leading to impaired mitochondrial function and reduced energy production through downregulation of STAT3 signaling [169].

Beyond tumor-cell-intrinsic effects, cysteine cathepsin inhibition can also reprogram immune and stromal responses. The activity-based inhibitor GB111-NH_2_, which targets CTSB, CTSL, and CTSS, alters macrophage polarization and lysosome-linked metabolic programs, thereby affecting proteolysis, inflammation, and stromal remodeling within the TME [136]. A major challenge in cathepsin inhibitor development is achieving sufficient selectivity. Many cathepsins share structurally similar active sites, particularly among CTSL-, CTSS-, and CTSK-like peptidases, enabling compensatory protease activity when a single enzyme is inhibited. This functional overlap may reduce the effectiveness of single-target inhibition approaches. However, preclinical studies have shown that selectively targeting individual cathepsins can modulate tumor-associated processes. In particular, inhibition of cathepsin B has been reported to reduce tumor invasion and related proteolytic activity in experimental cancer models [170]. Additionally, CTSZ/X has been proposed as a relevant target in tumor biology, with selective inhibitors demonstrating effects on cell adhesion and migration in preclinical settings [171]. Modulation of CTSB and CTSZ/X activity may influence tumor progression-related phenotypes, supporting the potential value of selective inhibition strategies in preclinical oncology models [172]. Therefore, rational multi-target inhibition strategies or combination therapies may be more effective than single-enzyme blockade.

An important limitation in translating lysosome- and cathepsin-targeted therapies from preclinical models to human PDAC is the presence of species-specific differences in cathepsin expression patterns, substrate specificity, and stromal biology. Several cathepsins display distinct expression profiles and functional redundancy between murine and human tissues, which may influence both tumor progression and therapeutic responses. In genetically engineered mouse models of PDAC, cathepsin activity is often strongly associated with inflammatory and stromal compartments, whereas human PDAC exhibits greater intertumoral heterogeneity and more complex spatial distribution of cathepsin expression across malignant, stromal, and immune-cell populations [108,173]. Moreover, differences in ECM composition, immune-cell infiltration, and desmoplastic architecture between mouse models and human tumors may alter lysosomal signaling dynamics and drug penetration. These translational discrepancies should be considered when interpreting preclinical efficacy data for cathepsin- or lysosome-targeting strategies in PDAC.

### 5.3. Cathepsin-Responsive Targeting Strategies, Theranostics, and Prodrug Systems

Elevated cathepsin activity in PDAC tumors and their TME has created opportunities to exploit these enzymes as proteolytic triggers for targeted drug delivery and diagnostic imaging. In these systems, cathepsin activity serves as an enzymatic switch that activates therapeutic payloads specifically within the tumor, improving drug penetration while reducing systemic toxicity. Multiple polymer conjugates, nanoparticles, and prodrug systems incorporating CTSB- or CTSS-cleavable linkers have been engineered to deliver gemcitabine and other anticancer agents preferentially to PDAC tissues [166,171,173,174,175,176,177,178,179,180,181,182,183,184,185,186]. Because CTSB is frequently overexpressed and enzymatically active in PDAC cells and their TME [94,113], it has been widely used as a proteolytic trigger in nanotherapeutic design. Peptide linkers such as GFLG motifs have been incorporated into gemcitabine prodrugs and ligand-targeted nanoparticles, including RGD- or uPAR-directed delivery platforms, enabling intracellular drug release following endocytosis and lysosomal processing [171,176]. Some constructs integrate self-reporting fluorescence activation, allowing real-time monitoring of enzymatic drug release and therapeutic engagement [169].

CTSE shows a highly PDAC-selective expression pattern, being upregulated early during pancreatic intraepithelial neoplasia (PanIN) progression and remaining elevated in invasive PDAC [128,187]. This has enabled the development of CTSE-activatable fluorescent probes for in vivo imaging and confocal endomicroscopy-based detection of neoplastic pancreatic lesions [86,188]. CTSE-responsive prodrug systems have also been explored, including enzyme-cleavable 5-aminolevulinic acid derivatives that generate localized photodynamic cytotoxicity after activation within the TME [167].

Cathepsin-dependent vulnerabilities can also be exploited to target stromal components. Magneto-mechanical activation of nanoparticles has been shown to induce lysosomal membrane permeabilization and cathepsin release in PDAC CAF models, leading to stromal cell death [189]. Spatially programmed combination delivery strategies have further demonstrated the potential of cathepsin-triggered systems to address multiple components of the TME simultaneously. For example, CTSB-triggered gemcitabine release combined with a perivascular PI3K inhibitor depot enhanced drug penetration, repolarized tumor-associated macrophages toward an M1-like phenotype, and improved antitumor immunity in orthotopic PDAC models [190].

Cathepsin-responsive technologies are also being developed as theranostic platforms, enabling simultaneous detection and treatment of tumors. Near-infrared probes capable of reporting protease activity in vivo [164], radiotherapeutic delivery systems incorporating cathepsin-cleavable linkers [179], and antibody-based targeting strategies that engage immune effector functions such as antibody-dependent cellular cytotoxicity (ADCC) have all been explored [191]. More recently, highly sensitive cathepsin-activatable fluorescence probes (including NIR-II designs) and multifunctional CTSB-responsive nanoDDS platforms have continued to expand the theranostic toolkit, supporting real-time mapping of protease activity to guide delivery strategies [192].

Despite strong biological rationale, therapeutic targeting of the lysosome–cathepsin axis in PDAC has so far produced limited clinical benefit. Available studies collectively suggest that this discrepancy reflects not failure of the pathway as a pathogenic driver, but rather the adaptive and highly context-dependent nature of lysosomal signaling in PDAC. Preclinical models consistently demonstrate that lysosomal inhibition disrupts autophagy-dependent metabolic fitness and sensitizes tumor cells to chemotherapy-induced stress [145,148]. However, these effects are often observed under experimental conditions that do not fully recapitulate the stromal complexity, hypovascularity, extracellular acidosis, and metabolic heterogeneity of human PDAC. Clinical studies with hydroxychloroquine further illustrate this limitation: although pharmacodynamic evidence of autophagy inhibition and improved pathological responses was observed in neoadjuvant settings [153], these effects did not translate into meaningful survival benefit in metastatic disease [152]. This suggests that lysosomal dependency may be greatest in earlier or therapy-stressed disease states, whereas advanced PDAC retains sufficient metabolic plasticity to compensate through alternative nutrient-scavenging pathways.

A similar limitation applies to cathepsin-directed approaches. While inhibition of CTSB, CTSL, or CTSS reduces invasion, metastatic behavior, and tumor growth in experimental models [159,165,166], therapeutic efficacy is constrained by substantial functional redundancy within the cathepsin network and by the dual role of these proteases in tumor cells and stromal or immune compartments. Consequently, broad inhibition may produce systemic or microenvironmental effects that are difficult to control, whereas highly selective inhibition may be circumvented through compensatory protease activity. Importantly, some of the most promising results have emerged not from complete protease blockade, but from exploiting elevated cathepsin activity for spatially controlled drug delivery and theranostic activation [179,186,190]. These strategies partially overcome the delivery barriers imposed by the PDAC TME and may provide a more selective means of exploiting proteolytic activity.

Overall, current evidence supports the lysosome–cathepsin system as a genuine therapeutic vulnerability in PDAC, but indicates that effective clinical targeting will likely require biomarker-guided and combinatorial approaches rather than stand-alone lysosomal inhibition. In particular, future strategies will probably need to simultaneously address adaptive lysosomal biogenesis, metabolic rewiring, stromal barriers, and immune suppression, which together limit the durability of proteolytic-axis-directed therapies.

### 5.4. Biomarkers and Monitoring

Systemic and local measurements of extracellular cathepsin activity have been investigated as potential biomarkers in PDAC. Cathepsins such as CTSB and CTSL have been detected in patient biofluids, supporting their potential use in biomarker and targeting strategies (see Table 3) [102,107,193,194]. Cathepsins, including CTSB, CTSL, CTSS, and CTSH have also been identified in EUS-FNA samples from PDAC lesions, although current studies have not established clear correlations between expression levels and disease stage [195]. Circulating cathepsins may also serve as minimally invasive biomarkers. CTSB has been proposed as a urinary biomarker, and circulating CTSB and CTSL levels are elevated in pancreatic cancer compared with controls, with plasma CTSL associated with advanced disease and poorer overall survival [102,193,194].

Among cathepsins, CTSE shows the strongest disease enrichment. CTSE expression is markedly elevated in PDAC tissue and pancreatic juice compared to benign pancreatic conditions, supporting its diagnostic potential [128]. Additional studies have confirmed CTSE as a promising target for both imaging and therapeutic strategies in pancreatic cancer [196,197]. Activatable fluorescent probes that report CTSE activity enable in vivo detection of neoplastic and precursor lesions, linking protease activity to early tumor biology [86,188]. Importantly, CTSD has also been included in analytically validated serum multi-analyte signatures for early-stage PDAC, improving diagnostic discrimination beyond CA19-9 alone and highlighting the translational potential of protease-based liquid biopsy approaches [198].

In contrast, CTSB and CTSL are less disease-specific but more strongly associated with tumor aggressiveness. Elevated circulating CTSL has been linked to advanced disease stage and reduced survival [199], while elevated CTSB expression in PDAC tumor tissue correlates with tumor progression and stem-like phenotypes [103,114]. However, circulating cathepsin levels reflect contributions from tumor cells, stromal cells, and immune populations, which may limit specificity. Therefore, activity-based assays that quantify the active protease fraction, especially when combined with longitudinal sampling, may improve clinical interpretability compared with static abundance measurements alone [200].

Emerging evidence also suggests potential roles for additional family members. A Mendelian randomization analysis across digestive system tumors identified CTSF as a genetically supported exposure associated with pancreatic cancer risk, indicating possible involvement in disease susceptibility rather than tumor progression [127].

Collectively, current evidence supports a compartment-aware biomarker model in which CTSE is most informative for early detection, while CTSB and CTSL more strongly reflect tumor aggressiveness and disease dynamics, and CTSF may relate to inherited susceptibility. Integrating cathepsin measurements into multiplex biomarker panels, particularly those incorporating activity-based assays and longitudinal monitoring, may enhance the utility of protease biomarkers within emerging liquid-biopsy frameworks for PDAC.

**Table 3:** Cathepsin-Related Liquid Biopsy Readouts in PDAC.

Sample Type	Analyte(s)	Intended Application	Key Finding	Ref.
Urine	Cathepsin B (among candidate urinary biomarkers)	Non-invasive biomarker discovery	Urinary biomarker profiling identified tissue–type–specific candidates for upper GI cancers, including markers relevant to pancreatic cancer.	[193]
Serum/liquid biopsy	Enzymatic activity panel (arginase, MMP-1/3/9, cathepsins B & E, uPA, neutrophil elastase)	Early detection/screening concept	Multiplex enzymatic “signature” proposed for potential early detection of pancreatic cancers in liquid biopsies.	[107]
Serum (circulating levels)	Cathepsins D, B, L	Prognosis/malignant progression	Circulating cathepsins are reported as markers associated with malignant progression.	[194]
Plasma	Cathepsin L	Prognosis	Plasma CTSL evaluated as a potential prognostic marker in pancreatic cancer.	[102]
Pancreatic juice	Cathepsin E	Diagnostics	CTSE levels are significantly elevated in pancreatic juice from PDAC patients compared to benign pancreatic disease; high diagnostic specificity	[128]
Serum (circulating levels)	Cathepsin B	Prognosis	Increased CTSB levels correlate with tumor burden and invasive phenotype; associated with aggressive disease biology	[103]

### 5.5. Translational Challenges and Therapeutic Limitations of Targeting the Lysosome–Cathepsin Axis in PDAC

The available data collectively support the view that the lysosome–cathepsin system is a biologically relevant vulnerability in PDAC. Nevertheless, despite strong mechanistic rationale and consistently encouraging preclinical findings, therapeutic strategies targeting this axis have so far produced only limited clinical benefit. Rather than indicating a lack of biological relevance, this discrepancy likely results from the highly adaptive nature of lysosomal signaling, along with the profound metabolic, stromal, and spatial heterogeneity that characterizes PDAC. Representative studies illustrate both the promise and weaknesses of lysosome–cathepsin-directed strategies. In the randomized metastatic PDAC trial by Karasic et al. [152], HCQ was combined with gemcitabine/nab-paclitaxel and improved response-related endpoints, but did not significantly improve overall survival, suggesting that systemic autophagy inhibition was insufficient in advanced, metabolically heterogeneous disease. The main advantages of this study were its clinical relevance and the use of a standard chemotherapy backbone. Limitations included the nonspecific mechanism of HCQ, uncertain intratumoral drug exposure, incomplete assessment of lysosomal target inhibition within tumor tissue, and the advanced disease setting. In contrast, the neoadjuvant study by Zeh et al. [153] allowed pathological and pharmacodynamic assessment and showed improved histopathological responses, supporting the biological activity of HCQ-based combinations in earlier-stage disease. However, the absence of a clear survival benefit indicates that autophagy inhibition alone is unlikely to overcome PDAC resistance. More recent ERK/autophagy inhibition strategies, including LY3214996 plus HCQ, were mechanistically rational but showed minimal clinical activity, emphasizing that HCQ may be too weak or nonspecific to fully suppress therapy-induced lysosomal adaptation [155].

Preclinical cathepsin inhibitor studies provide proof-of-concept but also reveal important limitations. VBY-825 and related cathepsin inhibitors reduced tumor burden or invasive phenotypes in experimental models [169], but many studies relied on simplified cell culture systems, subcutaneous tumors, or tumor-volume readouts that do not fully capture human PDAC stromal density, hypovascularity, immune exclusion, and protease redundancy. Similarly, cathepsin-responsive prodrugs and nanoparticles offer improved spatial selectivity by exploiting protease activity for local drug release, but most remain preclinical and depend on heterogeneous intratumoral cathepsin activity, efficient tissue penetration, and validated pharmacodynamic cleavage readouts. These examples suggest that the proteolytic axis remains therapeutically exploitable, but probably not as a stand-alone target.

A principal limitation of lysosome-targeting approaches is that inhibition of autophagy or lysosomal function rarely leads to sustained suppression of tumor metabolic fitness. CQ- and HCQ-based therapies effectively disrupt late-stage autophagy under experimental conditions and can sensitize PDAC cells to chemotherapy-induced stress by increasing oxidative damage and lysosomal membrane instability [162]. However, clinical studies have repeatedly shown that pharmacodynamic evidence of autophagy inhibition does not necessarily translate into durable therapeutic responses or meaningful survival benefit [164].

Several factors likely contribute to this limited efficacy. PDAC cells exhibit remarkable metabolic plasticity and can compensate for impaired lysosomal degradation through alternative nutrient acquisition pathways, including macropinocytosis, stromal metabolite scavenging, and rewiring of lipid and amino acid metabolism [35]. In addition, lysosomal stress induced by pharmacologic inhibition may paradoxically activate TFEB-dependent transcriptional programs that restore lysosomal biogenesis and autophagic capacity, enabling tumor adaptation despite ongoing therapeutic pressure. This adaptive response is especially relevant in KRAS/MAPK-driven tumors, where lysosomal signaling is closely integrated with stress-response and metabolic-survival pathways.

At the same time, the PDAC TME imposes major pharmacologic barriers that substantially limit effective target inhibition in vivo. Dense desmoplastic stroma, poor vascularization, elevated interstitial fluid pressure, and heterogeneous perfusion collectively impair intratumoral drug delivery [10]. These limitations are particularly relevant for lysosomotropic agents such as CQ and HCQ, whose intracellular accumulation depends heavily on extracellular pH gradients and local tissue architecture [156]. Hypoxic and acidic tumor regions may therefore reduce effective lysosomal drug trapping and contribute to incomplete pathway suppression within malignant cells. As a result, therapeutic activity observed in cell culture systems and murine models may overestimate the degree of lysosomal inhibition that can realistically be achieved in human PDAC.

Direct inhibition of cathepsins faces a distinct but closely related set of translational challenges. Experimental studies consistently show that inhibition of CTSB, CTSL, CTSS, and related proteases can reduce invasion, ECM remodeling, metastatic behavior, and tumor growth [159,160]. However, the cathepsin network is characterized by substantial functional redundancy, overlapping substrate specificity, and context-dependent biological activity. Selective inhibition of a single protease may be insufficient because compensatory activity by other cathepsins can preserve proteolytic function. Conversely, broader inhibition strategies risk interfering with physiological lysosomal and immune-cell processes, limiting therapeutic selectivity and increasing the likelihood of off-target effects.

The dual role of cathepsins in both malignant and stromal compartments further complicates therapeutic targeting. In PDAC, cathepsins contribute not only to tumor cell invasion and metabolic adaptation, but also to antigen processing, macrophage polarization, ECM turnover, and broader immune regulation [129]. Consequently, protease inhibition may suppress tumor-promoting pathways while unintentionally disrupting mechanisms required for effective antitumor immunity or tissue homeostasis. The immunological consequences of sustained lysosomal and cathepsin inhibition remain insufficiently understood, as suppression of tumor-associated proteolysis may enhance antitumor immunity in certain contexts, whereas excessive inhibition of lysosomal processing could impair antigen presentation and T-cell priming in dendritic cells and macrophages. Because cathepsins are integral components of MHC-associated antigen-processing pathways, broad inhibition strategies may produce context-dependent immunological effects that differ substantially between tumor and immune cell compartments. This issue may become particularly relevant when lysosome-targeting therapies are combined with immune checkpoint blockade, where preservation of effective antigen presentation is essential for therapeutic responsiveness. Therefore, future cathepsin-inhibition strategies should distinguish tumor-cell and stromal proteolysis from APC-associated antigen-processing functions, ideally using compartment-selective delivery, activity-based pharmacodynamic monitoring, or intermittent dosing schedules that avoid sustained suppression of dendritic-cell and macrophage antigen presentation.

Another important limitation is the imperfect translational fidelity of currently available preclinical PDAC models. Although genetically engineered mouse models and xenograft systems reproduce several key features of pancreatic tumor biology, they do not fully recapitulate the complexity of human stromal organization, immune heterogeneity, extracellular matrix composition, or vascular dysfunction. Species-specific differences in cathepsin expression patterns and substrate utilization may further influence therapeutic responses. As a result, preclinical efficacy data for lysosome- or cathepsin-targeted therapies should be interpreted cautiously, particularly when evaluating strategies intended to modulate stromal or immune cell proteolysis.

Interestingly, some of the most promising translational results have emerged not from direct protease inhibition, but from exploiting proteolytic activity for spatially controlled drug delivery and theranostic activation. Cathepsin-responsive nanoparticles, enzyme-cleavable prodrugs, and activatable imaging probes use elevated protease activity as a tumor-selective activation mechanism rather than attempting to suppress lysosomal function globally [166]. Such approaches may partially overcome the delivery barriers imposed by the PDAC TME by enabling localized intracellular release of therapeutic payloads after lysosomal processing while limiting systemic toxicity. These findings suggest that conditional exploitation of protease activity may ultimately prove more feasible than complete pharmacologic blockade of the lysosomal proteolytic system.

Current evidence also suggests that only a subset of cathepsins may ultimately represent therapeutically actionable targets in PDAC. CTSB, CTSL, and CTSS appear to be the most consistently implicated in invasion, metabolic adaptation, immune modulation, and ECM remodeling, making them attractive candidates for combinatorial therapeutic strategies. In contrast, enzymes such as CTSE may prove more valuable as highly PDAC-selective diagnostic or theranostic biomarkers rather than direct pharmacologic targets, particularly given their restricted expression profile and utility in activatable imaging systems. Future research should therefore focus not only on developing more selective inhibitors, but also on defining context-specific protease functions, identifying predictive biomarkers of lysosomal dependency, and determining which patient subgroups are most likely to benefit from proteolytic-axis-directed therapies.

Taken together, these observations suggest that the limited clinical efficacy of lysosome–cathepsin–targeted therapies does not indicate failure of the pathway as a pathogenic driver, but rather highlights the challenge of therapeutically suppressing a highly adaptive and interconnected stress-response network within an exceptionally treatment-refractory tumor ecosystem. Future progress will likely depend on biomarker-guided patient stratification and rational combination strategies integrating lysosomal targeting with stromal modulation, metabolic inhibition, vascular normalization, MAPK pathway blockade, chemotherapy, or immunotherapy. In this context, the lysosome–cathepsin axis should be viewed less as an isolated therapeutic target and more as a dynamic regulatory system whose successful exploitation will require simultaneous disruption of multiple adaptive survival mechanisms operating within both tumor cells and the surrounding TME. Combination strategies should be selected according to the dominant resistance mechanism. Lysosomal inhibition may be most rationally combined with chemotherapy or MAPK-pathway blockade when treatment induces compensatory autophagy and TFEB activation. In tumors with severe desmoplasia and poor perfusion, stromal modulation or vascular normalization may be required before lysosomotropic drugs can achieve sufficient intratumoral exposure. In immune-excluded tumors, cathepsin targeting should be combined cautiously with immunotherapy, because inhibition of tumor-promoting proteolysis may improve immune access, whereas excessive suppression of APC lysosomal function could impair antigen presentation.

## 6. Conclusions and Future Perspectives

Recent advances have established the lysosome–cathepsin axis as an important regulator of PDAC progression and therapeutic adaptation. However, despite substantial mechanistic insight from preclinical studies, several key translational and biological questions remain unresolved. One major challenge is defining the context-dependent roles of individual cathepsins within distinct cellular compartments of the PDAC TME, including tumor cells, CAFs, TAMs, and other immune populations. The functional redundancy and compensatory activity among cathepsin family members further complicate therapeutic targeting and biomarker development. Another important unresolved issue concerns the dynamic regulation of lysosomal adaptation during therapy. Increasing evidence indicates that TFEB-driven lysosomal biogenesis, metabolic rewiring, and autophagy-associated stress responses can promote resistance to chemotherapy, MAPK inhibition, and lysosome-targeting agents. However, the temporal and spatial determinants of these adaptive programs within human PDAC remain poorly understood. Future studies integrating spatial transcriptomics, single-cell multi-omics, and functional imaging approaches will be essential to better define lysosomal heterogeneity and protease activity within the complex PDAC TME. In addition, species-specific differences in cathepsin expression and stromal organization between mouse models and human PDAC complicate the interpretation of preclinical findings and may partially explain the limited clinical success of lysosome-targeting strategies to date. Improved human-relevant experimental systems, including patient-derived organoids, ex vivo stromal co-culture platforms, and spatially resolved tumor models, will likely be required to enhance translational relevance.

Future therapeutic development should prioritize rational combination strategies rather than lysosome or cathepsin inhibition alone. Particularly promising directions include combining lysosome-targeting agents with stromal remodeling approaches, immune checkpoint blockade, metabolic therapies, or MAPK pathway inhibition to overcome adaptive resistance mechanisms. In parallel, advances in cathepsin-activatable imaging probes, activity-based biomarkers, and targeted drug delivery systems may facilitate patient stratification and real-time monitoring of proteolytic activity in vivo. Collectively, future progress in this field will depend on integrating mechanistic, spatial, immunologic, and translational perspectives to better define when and how the lysosome–cathepsin axis can be therapeutically exploited in PDAC. Rather than representing a single targetable pathway, the lysosome–cathepsin network should increasingly be viewed as a dynamic and context-dependent regulatory system whose vulnerabilities may only emerge through combination-based and microenvironment-informed therapeutic strategies.

## Data Availability

Data sharing is not applicable.

## Abbreviations

The following abbreviations are used in this manuscript:

APC	Antigen-presenting cell
ATG	Autophagy-related protein
CAF	Cancer-associated fibroblast
CQ	Chloroquine
ECM	Extracellular matrix
HCQ	Hydrochloroquine
MHC	Major histocompatibility complex
MMP	Matrix metalloproteinase
NET	Neutrophil extracellular trap
NK	Natural killer cell
PDAC	Pancreatic ductal adenocarcinoma
PSC	Pancreatic stellate cell
TAM	Tumor-associated macrophage
TFEB	Transcription factor EB
TME	Tumor microenvironment
uPA	Urokinase-type plasminogen activator
